# A Hybrid Sensing Approach for Pure and Adulterated Honey Classification

**DOI:** 10.3390/s121014022

**Published:** 2012-10-17

**Authors:** Norazian Subari, Junita Mohamad Saleh, Ali Yeon Md Shakaff, Ammar Zakaria

**Affiliations:** 1 School of Electrical & Electronic Engineering, Universiti Sains Malaysia (USM), Engineering Campus, 14300 Nibong Tebal, Penang, Malaysia; E-Mail: aziansubari@ump.edu.my; 2 Centre of Excellence for Advanced Sensor Technology (CEASTech), Universiti Malaysia Perlis (UniMAP), 0100 Kangar, Perlis, Malaysia; E-Mails: aliyeon@unimap.edu.my (A.Y.M.S.); ammarzakaria@unimap.edu.my (A.Z.)

**Keywords:** electronic nose, FTIR, honey classification, data fusion, pure honey

## Abstract

This paper presents a comparison between data from single modality and fusion methods to classify Tualang honey as pure or adulterated using Linear Discriminant Analysis (LDA) and Principal Component Analysis (PCA) statistical classification approaches. Ten different brands of certified pure Tualang honey were obtained throughout peninsular Malaysia and Sumatera, Indonesia. Various concentrations of two types of sugar solution (beet and cane sugar) were used in this investigation to create honey samples of 20%, 40%, 60% and 80% adulteration concentrations. Honey data extracted from an electronic nose (e-nose) and Fourier Transform Infrared Spectroscopy (FTIR) were gathered, analyzed and compared based on fusion methods. Visual observation of classification plots revealed that the PCA approach able to distinct pure and adulterated honey samples better than the LDA technique. Overall, the validated classification results based on FTIR data (88.0%) gave higher classification accuracy than e-nose data (76.5%) using the LDA technique. Honey classification based on normalized low-level and intermediate-level FTIR and e-nose fusion data scored classification accuracies of 92.2% and 88.7%, respectively using the Stepwise LDA method. The results suggested that pure and adulterated honey samples were better classified using FTIR and e-nose fusion data than single modality data.

## 1. Introduction

South East Asia, including Malaysia, is rich in natural forest resources such as honey. Honey is a viscous, supersaturated sugar solution derived from nectar gathered and modified by honeybee (*Apis dorsata*). According to the European Union (EU) regulations, the food *Codex Alimentarius* and various other international honey standards, “honey stipulates a pure product that does not allow for the addition of any other substance”. Currently, there is high market demand on pure honey. This has resulted in increased sales of adulterated honey claimed as pure honey by irresponsible parties. Many manufacturers have started to add variants of sugar in pure honey so that it has become difficult to differentiate pure honey samples from adulterated ones.

Various analytical procedures can be employed to determine food product authenticity such as Liquid Chromatography-Mass Spectrometry (LC-MS), Gas Chromatography-Mass Spectrometry (GC-MS), UV-Visible (UV-VIS) Spectrometry, High Performance-Liquid Chromatography (HPLC), isotopic analysis and deoxyribonucleic acid (DNA)-based analysis [1–9]. These analytical techniques are useful and accurate, but they have drawbacks such as being time-consuming and requiring highly skilled operators to perform the corresponding chemical separation processes [10].

This paper presents rapid assessment of honey purity using electrical aroma sensors, also known as e-noses, and FTIR. An e-Nose uses an association of several sensitive elements on which volatile compounds get bonded [11]. The adsorption induces an alteration of the electrical signals of these compounds. E-noses have been employed for various purposes such as assessment of melon and blueberry maturity [12,13] and sorting of fruits and vegetables according to their variety [14,15]. For FTIR, absorption bands in the mid infrared or near infrared range due to molecular vibrations can be detected [10]. FTIR has been widely employed for characterization of food products such as for assessment of sugar content, and detection of edible oil and apple juice adulteration [16–19].

This work presents various classical techniques to detect and discriminate adulteration in honey samples. This involves work performed to evaluate the potential of the Principal Component Analysis (PCA) features selection technique, and classification of honey using PCA and Linear Discriminant Analysis (LDA) methods based on e-nose, FTIR and the fusion of these two datasets. Classification accuracies from honey classifiers based on the various datasets have been compared to investigate the feasibility of using combined datasets.

## 2. Material and Methods

### 2.1. Sample Preparation

Ten different brands of pure Tualang honey were purchased from the local market (three different batches of each particular honey). The purity of these honey products were validated using a UV-VIS spectrometer to measure the scavenging ability of antioxidants towards the stable 1,1-diphenyl-2-picrylhydrazyl (DPPH) free radical, as carried out by previous researchers in the literature [20,21]. The performed validation test revealed that all the Tualang honey used in this work had DPPH values ranging from 32.25% to 73.20%. Previous work by Khalil *et al.* had reported that the DPPH scavenging percentage of various pure Tualang honeys ranged from 35.12% to 75.13% [22]. Hence, the DPPH values obtained from the Tualang honey samples used in this work are within the range reported by Khalil *et al.* This validated that all the honey samples used in this research work could be verified as pure.

In this work, two types of organic sugar solution; beetroot sugar obtained from Grafschafter Krautfabrik (Meckenheim, Germany) and cane sugar obtained from Lyle Golden Syrup (Bristol, United Kingdom), were used for preparation of adulterated honey samples. Table 1 lists all pure honey, sugar samples and adulterated honey used in this experiment with their respective labelling.

Three bottles of each pure honey product were purchased. Out of each bottle, three 5 mL samples were taken, hence producing nine samples for each honey product. As for adulteration samples, each pure honey product was prepared by mixing honey with cane sugar or beetroot sugar in different concentrations of 20%, 40%, 60% and 80%, as illustrated in Table 2. Ten samples were produced for each concentration of adulteration honey. In total there were 172 samples of pure honey, pure sugar and adulterated honey. Each pure and adulterated honey was replicated five times, while pure sugar (beetroot and cane sugar) ones were replicated ten times. This was done to verify that all the data were from the same product.

### 2.2. Electronic Nose (E-Nose) Measurements

A number of previous articles had proven that pattern recognition techniques can be useful in agricultural applications when e-nose technology is applied [23–26]. In this classification work, a Cyrano Sciences Cyranose 320 e-nose was used. It is a portable system from Smith Detection™ (Pasadena, CA, USA) consisting of 32 individual polymer sensors blended with carbon black composite. The polymer sensors are potentiometric sensors configured as an array. They are made up of various conducting polymers to sense a variety of vapour mixtures. When the polymer sensors are exposed to honey vapour, each sensor absorbs its specialized vapour and swells like a sponge. During swelling, the distance between the conductive carbon black particles increases and hence, increasing the resistance of the composite [27,28]. This type of e-nose polymer sensor had been employed for many applications, including quality control in food industry, plant disease detection and biomedical sample discrimination [24,29,30].

**Figure 1.**
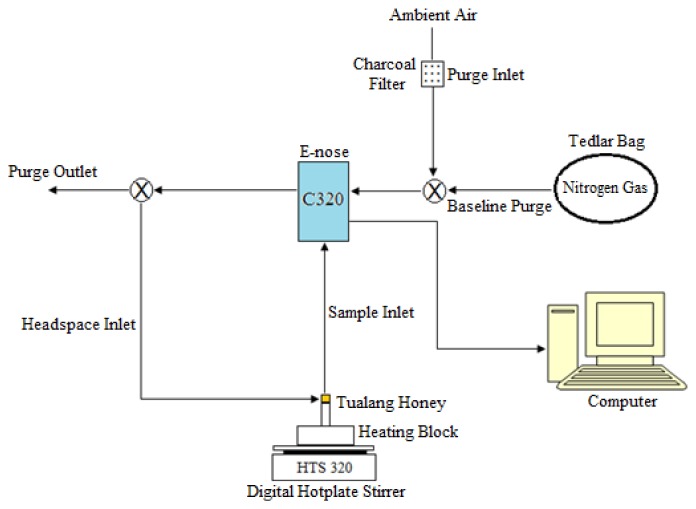
E-nose setup for headspace evaluation of honey, sugar and adulteration sample.

The e-nose setup used in this work was as illustrated in Figure 1. The filter used was made of activated carbon granules and had a large surface area, making it effective in removing a wide range of volatile organic compounds and moisture in the ambient air. The setting on the sniffing cycle of the Cyranose 320 (C320) as indicated in Table 3. In this work, preliminary experiments were performed to determine the optimal experimental setup for the purging, baseline purge and sample draw durations.

Before measurement is made, each sample was placed in a heating block and heated-up to generate sufficient headspace volatiles. The temperature of the sample was controlled at 50 ± 1 °C during the headspace collection. Five mL of each sample was drawn using a syringe and kept in a 13 mm × 100 mm test tube sealed with a silicone stopper. When the sensors are exposed to a vapour-phase analyte, the sensor matrix will swell and increase in volume causing an increase in resistance because the carbon black pathways through the material are broken. The changes in resistance across the array were captured as digital patterns, representing test smells. The combination of resistances from all the sensors should provide adequate information for the honey adulteration detection task and hence, could allow for qualitative and quantitative assessments of complex solutions.

### 2.3. Fourier Transform Infrared Spectroscopy Measurement (FTIR)

FTIR has been used extensively for various applications [31–35]. In this work, FTIR spectral measurements were gathered at room temperature of 27 °C using a Perkin Elmer 1600 FTIR Spectrometer (Waltham, MA, USA). This FTIR Spectrometer is equipped with a ATR crystal having coverage of the 4,000 to 650 cm^−1^ spectral region. The spectral measurements were performed against a background baseline of distilled water and presented in total attenuation units. The crystal surface was cleaned with distilled water and dried with tissue paper (Kimberly-Clark, Selangor, Malaysia) after the measurement of each sample. The background spectrum obtained from the first measurement was verified through the spectrum waveform to ensure the surface of the crystal was cleaned and free from previous sample residue. Then, a small drop of honey sample was placed on the crystal using a syringe and measurements were taken. Each sample was scanned four times and the measurements were averaged.

The spectral data were processed using FTIR spectroscopy spectrum software version 5.0.1 by Perkin Elmer for baseline correction, smoothing and normalization. Baseline correction is a process of removing background noise by eliminating the dissimilarities between spectra due to shifts in baseline. Smoothing is essential to reduce high frequency instrumental noise and enhance information content of a spectrum. Normalization of spectra eliminates the path length variation and reduces the differences between measurements of a single sample. Usually the spectra are normalized to the most intense band or at the same integrated intensity within a given spectral region [36].

### 2.4. Data Analysis

The following subsections explain the methods of data pre-processing employed prior to classification of pure honey.

#### 2.4.1. Preprocessing of Electronic Nose Data

E-nose data acquired by the Cyranose 320 is a set of relative changes in the resistances of the polymer composites sensors during exposure to the gas of interest. Firstly, all the e-nose data were pre-processed automatically in MATLAB using the fractional measurement technique known as baseline manipulation. Using this technique, new sets of pre-processed data, *S_frac_* were obtained based on:

(1) $$S_{\text{frac}}=[{\text{S}}_{\text{t}}-{\text{S}}_{0}]/{\text{S}}_{0}$$

where *S_0_* is the minimum value taken during baseline purge with ambient air and *S_t_* is the sensor response obtained in a sample draw. As each sensor has large varying levels of response, this equation gives a unit response for each sensor array according to its baseline. Therefore, the effect of temperature, humidity and temporal drift could be minimized [11].

Secondly, the pre-processed data were normalized based on the minimum and maximum values of each data using the following equation:

(2) $$S_{\text{norm}}=[S_{\text{frac}}-S_{\min}]/[S_{\max}-S_{\min}]$$

Using Equation (2), the data were then bounded within 0 and 1. The purpose of using normalized data in this work was to investigate its use in enhancing the classification accuracy of honey detection in comparison to using raw data, when various statistical classification methods were employed as the classifier. This comparison is important as classification capability depends on the range of input data for input-output mapping task.

#### 2.4.2. Pre-Processing of FTIR Spectra

**Figure 2.**
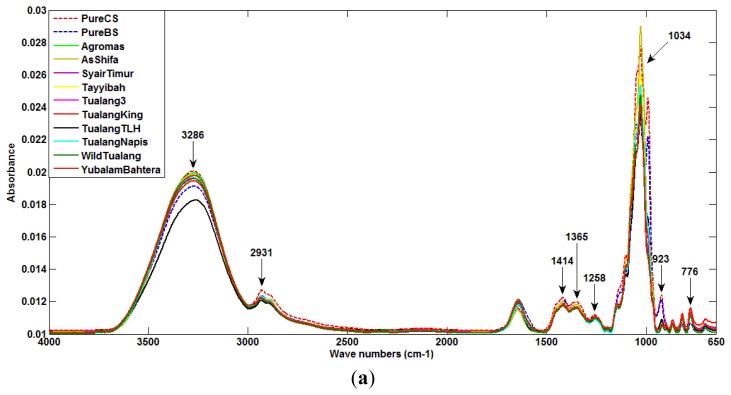

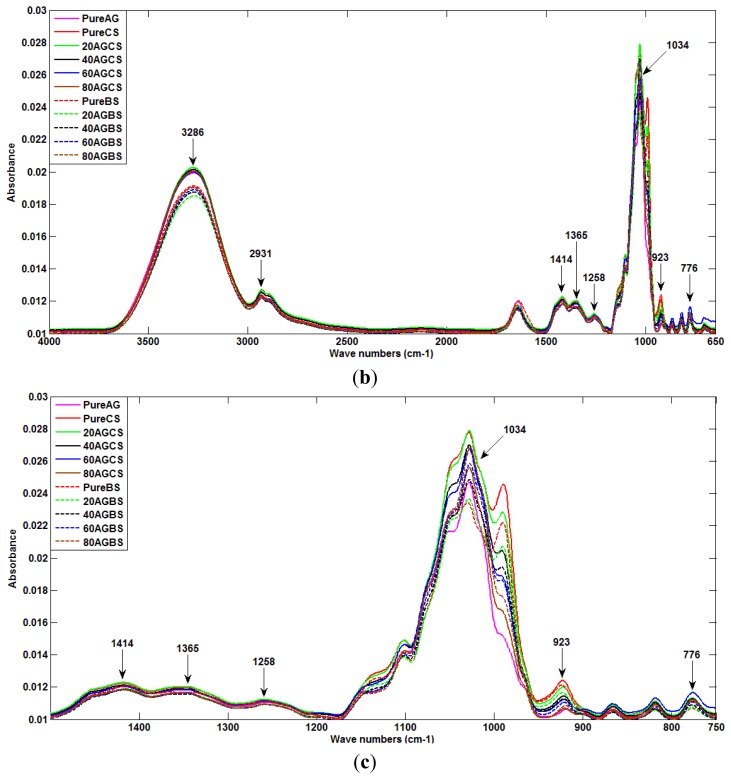
(**a**) All Pure Tualang honey and sugar solutions for full spectra range (650 cm^−1^ to 4,000 cm^−1^). (**b**) All AG honey sample for full spectra range (650 cm^−1^ to 4,000 cm^−1^). (**c**) AG samples for spectra range from 750 cm^−1^ to 1,500 cm^−1^.

Figure 2(a) to 2(c) show the spectra of different samples used as data for this research work. Figure 2(a) presents the ATR spectra for all pure Tualang honey and pure sugar solution (beetroot and cane sugar) for the entire spectrum with the corresponding band assignments. There are twelve peaks in the plots of each sample data. They show molecular vibration patterns of each honey. As can be observed, spectra of pure honey and pure sugar solution show absorbance bands at 776, 923, 1,034, 1,258, 1,365, 1,414, 2,931, and 3,286 cm^−1^.

Figure 2(b) shows the spectral pattern of all AG honey products, including pure samples and ones adulterated with beetroot sugar and cane sugar. A similar spectra pattern as for pure honey and pure sugar solutions can be seen for each adulterated honey product.

Figure 2(c) shows the zoomed-in spectral region between 750 cm^−1^ and 1,500 cm^−1^ for all AG samples. This region corresponds to the attenuation or absorption of the three major sugar constituents of honey; fructose, glucose and sucrose [37]. The 750 to 900 cm^−1^ region is the anomeric region, showing the characteristic saccharide absorptions [37]. The highest peak at 1,034 cm^−1^ is assigned to the C-O stretching band. The peak at 1,414 cm^−1^ is assigned to the carbohydrateC-H stretching band. According to the data provided in Figure 2(c), the absorbance values for glucose of pure honey, and pure beetroot sugar and sugar cane at 1,034 cm^−1^ are about 0.0245, 0.023 and 0.028, respectively. The different values suggest the feasibility of honey purity detection.

**Figure 3.**
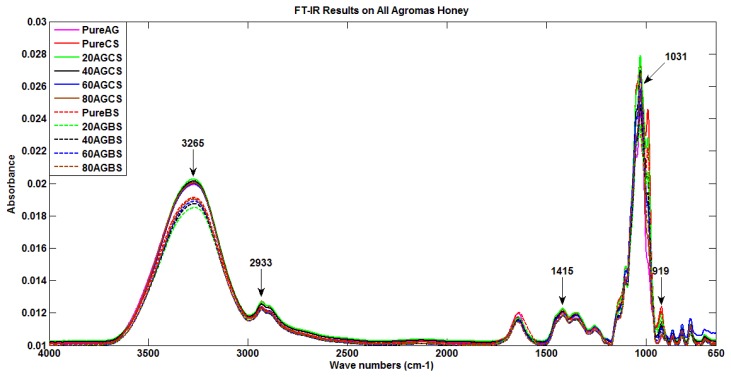
All Agromas honey brand sample with the corrected peak height.

Few works have reported on features extraction from FTIR spectra, such as the features wavelength method, comparing the standard deviation of samples and the derivative investigation [31–33]. This work aims to adopt a new feature selection approach based on a few methods commonly applied to medical data such as corrected peak height, corrected area and area under spectrum [32,33,35,36]. In this work, corrected peak height has been proposed as the feature selection technique to identify the authenticity of honey. The selection of the features based on the functional class contain in honey samples used. Based on the plot in Figure 3, it can be seen that there are five highest peaks with absorption values larger than 0.012. Hence, only five obvious peaks as depicted in Figure 3 are used as salient features for detection of honey adulteration. The selected features are:

- Corrected peak height at 919 cm^−1^
- Corrected peak height at 1,031 cm^−1^
- Corrected peak height at 1,415 cm^−1^
- Corrected peak height at 2,933 cm^−1^
- Corrected peak height at 3,265 cm^−1^

#### 2.4.3. Statistical Analysis of Data

Few articles have reported the success of PCA and LDA techniques at discriminating data into appropriate clusters or groups [37–42]. Hence, this work attempts to investigate the potentials of both PCA and LDA techniques at classifying pure and adulterated honey. Both approachws were executed using MATLAB 7.0.

PCA is a statistical technique relying on a linear projection of multidimensional data onto coordinates based on maximum variance and minimum correlation for feature extraction [23,24,43]. It transforms the original set of features into a smaller subset of linear combinations, called principal components (PCs) that account for the most variance in the original dataset [44]. Selected PCs are normally uncorrelated variables obtained by multiplying the original correlated variables with a pre-calculated eigenvector. The eigenvalues of PCs are the measurements of their associated variance. The first PC explains the largest percentage of the total variance, usually more than 80%, and so forth. A plot of the first two PCs can be used to determine whether distinct data clusters exist for pattern recognition. In this work, the PCA technique was used to pre-process data corresponding to honey samples. Then, plots of the first two PCs were observed to determine the existence of distinct clusters for the task of classifying pure or adulterated honey.

LDA is another statistical method used to distinct pure and adulterated honey samples. LDA is a study of random variable or random sample emanating from different groups, to allocate a sample of unknown origin to an appropriate group [38,39]. It is a supervised exploratory data analysis. It transforms the original variables into new variables by deriving linear combinations of independent variables that help to discriminate between prior defined groups. The discrimination is accomplished by maximizing between-group variances, relative to within-group variance. This way, the misclassification error is minimized. In this work, the honey datasets were divided into training and validation sets by randomly subdividing the available pattern vectors into two equal sets (*i.e.*, 50% training and 50% validation) as done by a previous work [44]. Then, correct classification accuracies based on the Direct and Stepwise LDA methods were investigated and compared. In the Direct LDA method, all independent variables were considered and analyzed simultaneously. The Stepwise method involved variable selection using Wilk's Lambda. Only the lower values were selected in the equation. The selected values were counted for F-statistic whose values must be in the range of the F-to-remove and F-to-enter. Following a few research works in literature, the values chosen for F-to-remove and F-to-enter were 2.71 and 2.84, respectively [45]. Fisher linear discriminant function and leave-one-out were also applied in both analyses.

#### 2.4.4. Data Outliers

An outlier is defined as an observation that “appears” to be inconsistent with other observations in the data set [46,47]. An outlier originates from the same statistical distribution as the other observation in a set of data. Outliers normally occur due to incorrect experimental procedure. Noise in the system and drift effects in the experiments are also among the main causes of outliers. If a data value has low probability, this indicates bad data. If it can be determined that an outlier point is in fact erroneous, then the outlier value should be deleted from the analysis [48]. Results of experiments are expected to show some improvement once outliers are removed from the original data. In this work, seven outliers were found in the E-nose and FTIR data and hence, these values were removed.

### 2.5. Data Fusion

Data fusion is a technique of combining data from multiple sensors or from different electronic systems. In literature, this technique has been shown to be able to simplify interpretation of experimental data and improve system performance, compared to using single modality [49–56]. Usually, the key to a successful fusion method is dependent upon complementary information provided by the additional sensor [57].

Fusion methods can be categorized as Low-Level Fusion (LLF), Intermediate-Level Fusion (ILF) and High-Level Fusion (HLF). This paper aims to investigate the feasibility of using LLF and ILF for detection of honey adulteration. The following subsection discusses about these two fusion techniques.

#### 2.5.1. Low-Level Fusion (LLF)

**Figure 4.**
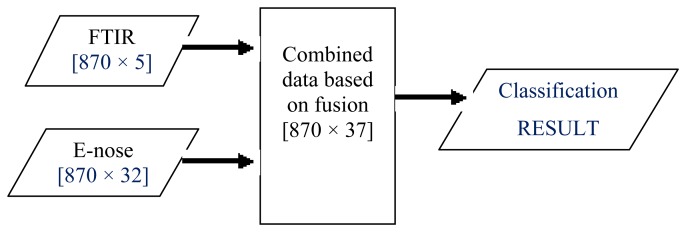
Low-level fusion scheme.

LLF involves combining two or more sensor outputs to create a single signal. In the literature, this fusion level had been successfully used in grading white grapes, discrimination of standard fruit and image enhancement [10,50–52]. As this fusion technique does not require different modalities to have the same number of features, this work simply concatenated or fused pre-processed data from e-nose and FTIR as illustrated in Figure 4. The fusion of FTIR (five features) and e-nose data (32 resistance values) gave a total of 37 signals for the honey classification task.

#### 2.5.2. Intermediate-Level Fusion (ILF)

**Figure 5.**
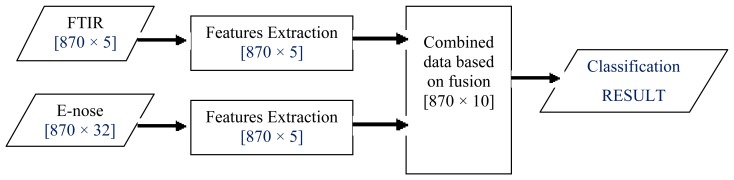
Intermediate-level or feature-level fusion scheme.

ILF, also known as feature-level fusion, first involves feature extraction onto each source of data (FTIR and e-nose). Then, ILF is accomplished by a simple concatenation of the feature sets obtained from multiple information sources [52–55]. Let FTIR data be ***X*** and e-nose data be ***Y***, denoted as feature vectors (***X****_f_* and ***Y****_f_*) representing the information extracted via two different sources. These features are then fused by concatenating them into a single vector for classification task as illustrated in Figure 5.

In this work, five salient features from each FTIR dataset were extracted, as explained in Section 2.4.2. As for e-nose data, feature extraction based on the PCA technique was performed on each set. This resulted in five PCs from each e-nose dataset. Hence, when FTIR and e-nose data were fused, a total of 10 features were obtained and used as inputs to the honey classification system.

Once data had been prepared, classifiers based on LDA were first separately trained with the e-nose and FTIR datasets (*i.e.*, without fusion). Then, the third classifier was trained with fusion datasets using LDA classification method. All the trained classifiers were cross-validated by employing the leave-one-out method based on the available data to validate their classification accuracies. These procedures were applied on both raw and normalized data to compare the classification accuracies of classifiers trained on various datasets.

## 3. Results and Discussion

### 3.1. E-nose Result

**Figure 6.**
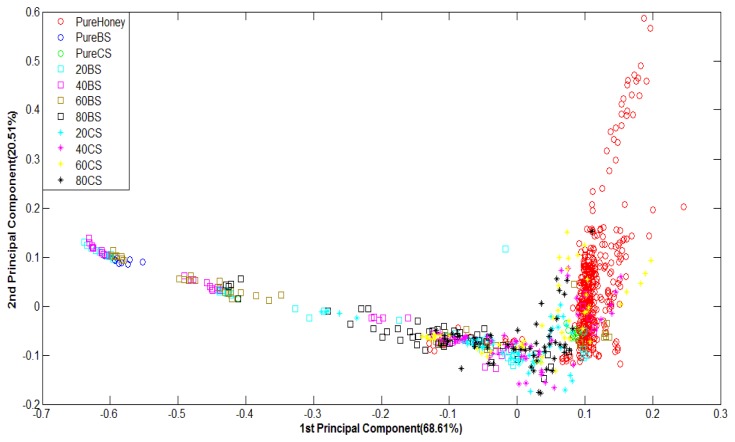
PCA plot of normalized E-nose data.

Figure 6 shows PCA classification results. It can be observed that PCA is less effective at discriminating e-nose responses of various honey odours. The data from pure honey seem rather properly clustered, but other groups of honey and pure sugar are scattered every where. Therefore, PCA has not been able to properly group most types of honey although the total variances for the first two principal components are rather high; 99.75% for raw data and 89.12% for normalized data.

**Figure 7.**
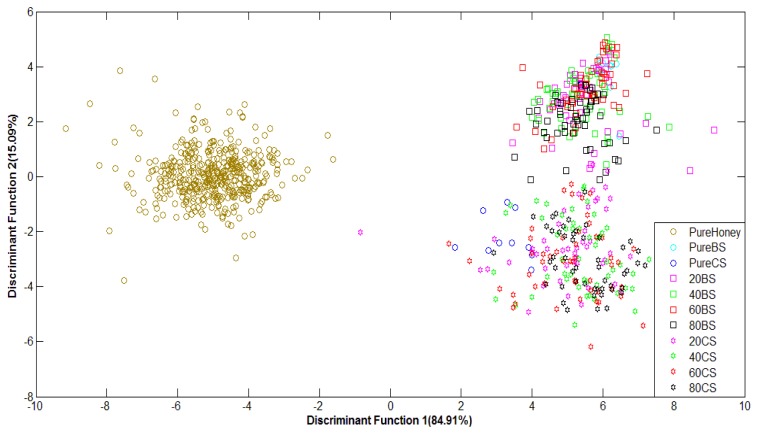
LDA plot of normalized E-nose data.

Figure 7 shows the classification plot of pure honey, adulterated honey and pure sugar based on normalized data using LDA as classifier. It can be observed that the groups of pure and adulterated honey are distinctly separated, although the groups of adulterated samples are overlapping. This is an improvement in classification from the PCA technique.

Similar behaviour is also observed for honey classification based on raw data. After validation, raw data using Stepwise LDA achieved the highest classification accuracy of 76.5%, while normalized data achieved a highest accuracy of 74.9% using the Direct LDA method. Based on visual comparison, it can be seen that the LDA technique is able to separate the clusters of pure honey, adulterated honey and pure sugar solution. Hence, the statistical analysis reveals that LDA is better than PCA at honey classification based on e-nose data. A comparison between the use of PCA and LDA showed both techniques have about the same execution speed, although PCA is easier to implement than LDA.

### 3.2. FTIR Result

**Figure 8.**
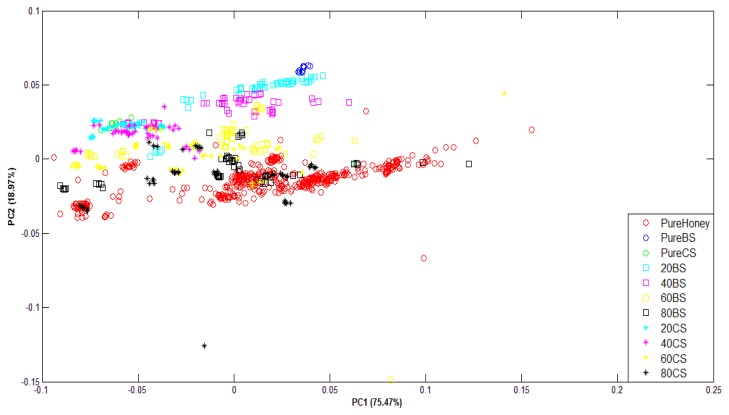
PCA plot of normalized FTIR data.

Figure 8 shows the PCA plots of normalized FTIR data. For FTIR data, the first two principal components of FTIR data accounted for 90.12% of the variance for raw data and 94.44% of the variance for normalized data. It can be observed that although the variance values are high and the groups are in clear sequence, they have not been well-clustered into adulterated and pure honey. This suggests that PCA technique has not been able to differentiate between adulterated and pure honey.

**Figure 9.**
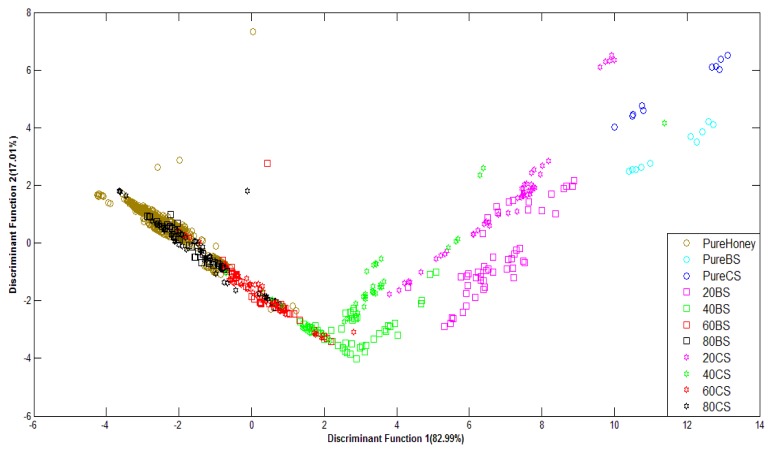
LDA plot of normalized FTIR data.

Figure 9 shows the performance of LDA classification of pure honey, adulterated honey and pure sugar solutions based on normalized FTIR data. The LDA technique has been able to separate different groups of honey in sequence, with slight overlapping. Classification based on normalized FTIR data gives lower accuracy of 68.4% compared to the raw data with 88.0% classification accuracy. This is due to slight overlapping between pure honey and 80% adulterated honey results. Overall, the supervised LDA technique shows better classification for both raw and normalized data in comparison to the PCA technique.

### 3.3. Low-Level Fusion (LLF) Result

**Figure 10.**
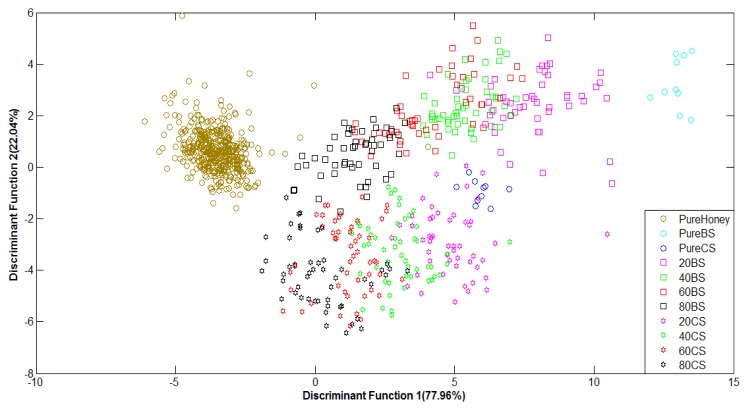
LDA plot of LLF normalized data (e-nose and FTIR).

As the previous work stages has revealed that LDA method is more robust than PCA at classification of adulterated and pure honey, our subsequent investigation based on fused data only focused on the LDA method. Figure 10 shows the plot of normalized data (e-nose and FTIR data) with classification score of 92.2% using the Stepwise LDA method. It can be seen from the figure that using normalized fusion data, pure and adulterated honey groups are clearly separated. Similar behavior is also observed with raw data, but only 91.7% of correct classification is achieved. This suggests that higher classification accuracy can be obtained using normalized LLF data.

### 3.4. Intermediate-Level Fusion (ILF)

**Figure 11.**
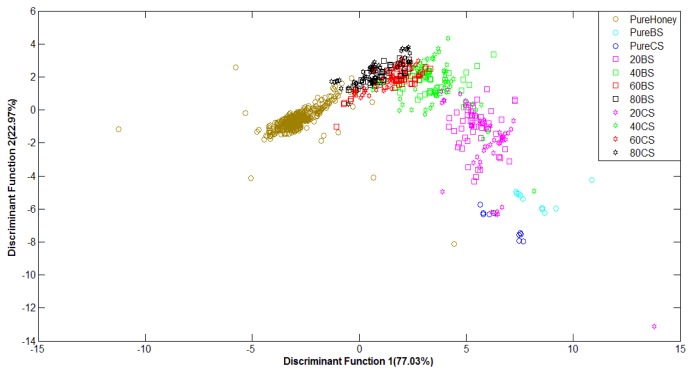
LDA plot of ILF norm data.

The result for honey classification using the normalized ILF data is as depicted in Figure 11. It can be observed from the figure that the main aim of classifying pure and adulterated honey is clearly accomplished as these two groups are distinctively separated. Classification based on the normalized ILF data gave a correct classification of 88.7%. This is obtained based on the Stepwise LDA method.

The results of classification based on raw ILF data also show that all the various types of honey have been grouped in sequence according to the types of honey purity with a little overlapping. With classification based on raw ILF data, the highest accuracy of 88.2% was obtained using the Stepwise LDA method. Therefore, it can be concluded that classification based on normalized ILF data is able to give higher accuracy than classification based on raw ILF data.

Overall, the results of pure or adulterated honey classification using e-nose, FTIR, LLF and ILF datasets revealed that the LDA method gave higher accuracy than PCA. As already explained, two LDA techniques—Direct and Stepwise—were used. Table 4 summarizes all LDA classification results for the training and validation data based on both, raw and normalized data (indicated as Norm Data in the table) for the Direct and Stepwise (after Wilk's lambda) LDA techniques. The highest accuracy values for each type of dataset are bold. Based on the validation accuracies, the classification results showed an improvement when the data of e-nose and FTIR were fused or combined using the LLF method. This is because fusion of both sensor features provides more salient information that further contributes towards better classification performances. Further comparison between LLF and ILF honey classifiers show that classifier based on LLF data is able to give higher accuracy than the ILF classifier.

## 4. Conclusions

In this research work, the classification performance of single modality based on either e-nose or FTIR data, and a method of fusion of e-nose and FTIR data at classifying honey (either pure or adulterated) was investigated. Five selected peaks in the FTIR spectra and thirty-two resistance values obtained from the e-nose system were used. The PCA was used as a data pre-processing method as well as a classifier, in comparison to the LDA method, focusing on the Direct and Stepwise techniques.

Overall analysis showed that LDA method was able to distinctively group the various honey samples better than the PCA technique. Honey classification using FTIR data gave higher accuracy than classification using e-nose data based on the LDA technique. Nonetheless, higher classification accuracies had been achieved using low-level and intermediate-level fusion methods compared to using any of the single modality data. Further investigation revealed that honey classifier based on LLF data was able to give higher classification accuracy than honey classifier based on ILF data. The results also showed that Stepwise LDA method gave higher classification accuracy than the Direct LDA method for fusion data. In summary, the work had shown the superior potential of fusion methods to assist human panels in classifying pure and adulterated honey. In the future, high-level fusion methods could be investigated as a comparison to LLF and ILF techniques in the classification of honey.

## Figures and Tables

**Table 1.** Description and abbreviation of honey and sugar samples used in the experiments.

**Product Type**	**Product Name**	**Product Label**	**Label for Honey + Beetroot Sugar (BS)**	**Label for Honey + Cane Sugar (CS)**
Pure Honey	Agromas	AG	AGBS	AGCS
Pure Honey	As-Shifa	AS	ASBS	ASCS
Pure Honey	Syair Timur	ST	TBS	TCS
Pure Honey	Tayyibah	T	TBS	TCS
Pure Honey	Tualang Tiga	T3	T3BS	T3CS
Pure Honey	Tualang King	TK	TKBS	TKCS
Pure Honey	Tualang TLH	TLH	TLHBS	TLHCS
Pure Honey	Tualang N'Apis	TN	TNBS	TNCS
Pure Honey	Wild Tualang	WT	WTBS	WTCS
Pure Honey	Yubalam	YB	YBBS	YBCS
Pure Sugar	Bahtera	BS	-	-
Pure Sugar	Beetroot Sugar	CS	-	-
Pure Sugar	Cane Sugar			

**Table 2.** Description of mixture for different samples of honey and sugar (BS or CS).

**Label**	**Percentage of Pure Honey**
AG,AS,ST,T,T3,TK,TLH,TN,WT or YB	100%
BS	0%
CS	0%
20BS or 20CS	80%
40BS or 40CS	60%
60BS or 60CS	40%
80BS or 80CS	20%

**Table 3.** E-nose Parameter Setting.

	**Cycle**	**Time (s)**	**Pump Speed**
**Sampling**	Baseline Purge	10	120 mL/min
**Setting**	Sample Draw	40	120 mL/min
	Idle Time	3	-
	Air Intake Purge	40	120 mL/min

**Table 4.** Summary of LDA classification results.

**Modality**	**Accuracy of Training Data (%)**				**Accuracy of Validation Data (%)**			
	
	Raw Data		Norm Data		Raw Data		Norm Data	
	
	Direct	Stepwise	Direct	Stepwise	Direct	Stepwise	Direct	Stepwise
E-Nose	73.0	72.8	69.1	70.3	74.9	**76.5**	74.9	71.9
FTIR	91.7	91.9	79.5	79.5	**88.0**	87.5	68.4	64.1
LLF	92.6	93.1	91.2	91.2	91.7	90.3	90.3	**92.2**
ILF	80.2	81.1	87.0	87.0	81.8	88.2	81.6	**88.7**
